# Genetics of Ocular Melanoma: Insights into Genetics, Inheritance and Testing

**DOI:** 10.3390/ijms22010336

**Published:** 2020-12-30

**Authors:** Natasha M. van Poppelen, Daniël P. de Bruyn, Tolga Bicer, Rob Verdijk, Nicole Naus, Hanneke Mensink, Dion Paridaens, Annelies de Klein, Erwin Brosens, Emine Kiliҫ

**Affiliations:** 1 Department of Ophthalmology, Erasmus Medical Center, 3015 GD Rotterdam, The Netherlands; n.vanpoppelen@erasmusmc.nl (N.M.v.P.); d.p.debruyn@erasmusmc.nl (D.P.d.B.); drtolgabicer@gmail.com (T.B.); n.naus@erasmusmc.nl (N.N.); d.paridaens@oogziekenhuis.nl (D.P.); 2 Department of Clinical Genetics, Erasmus Medical Center, 3015 GD Rotterdam, The Netherlands; a.deklein@erasmusmc.nl (A.d.K.); e.brosens@erasmusmc.nl (E.B.); 3 Department of Ophthalmology, University of Health Sciences Diskapi Training and Research Hospital, Ankara 06330, Turkey; 4 Department of Pathology, Erasmus Medical Center, 3015 GD Rotterdam, The Netherlands; r.verdijk@erasmusmc.nl; 5 Department of Pathology, Leiden University Medical Center, 2333 ZA Leiden, The Netherlands; 6 Department of Ophthalmic Oncology, The Rotterdam Eye Hospital, 3011 BH Rotterdam, The Netherlands; h.mensink@oogziekenhuis.nl

**Keywords:** ocular melanoma, genetics, uveal melanoma, iris melanoma, conjunctival melanoma, noninvasive testing

## Abstract

Ocular melanoma consists of posterior uveal melanoma, iris melanoma and conjunctival melanoma. These malignancies derive from melanocytes in the uveal tract or conjunctiva. The genetic profiles of these different entities differ from each other. In uveal melanoma, *GNAQ* and *GNA11* gene mutations are frequently found and prognosis is based on mutation status of *BAP1*, *SF3B1* and *EIF1AX* genes. Iris melanoma, also originating from the uvea, has similarities to the genetic makeups of both posterior uveal melanoma (UM) and conjunctival melanoma since mutations in *GNAQ* and *GNA11* are less common and genes involved in conjunctival melanoma such as *BRAF* have been described. The genetic spectrum of conjunctival melanoma, however, includes frequent mutations in the *BRAF*, *NRAS* and *TERT* promoter genes, which are found in cutaneous melanoma as well. The *BRAF* status of the tumor is not correlated to prognosis, whereas the *TERT* promoter gene mutations are. Clinical presentation, histopathological characteristics and copy number alterations are associated with survival in ocular melanoma. Tissue material is needed to classify ocular melanoma in the different subgroups, which creates a need for the use of noninvasive techniques to prognosticate patients who underwent eye preserving treatment.

## 1. Introduction

The first known description of uveal melanoma (UM), a specific form of ocular melanoma, dates from 1868, described by the German ophthalmologist and otolaryngologist Hermann Knapp [1]. Various subtypes based on cell type and pigmentation among other characteristics were later described in 1882 by Austrian ophthalmologist Ernst Fuchs. He also stated that enucleation was the treatment of choice, a treatment that is still used currently [2]. UM was a rare disease in that century; it still is, but the incidence is rising [3,4,5].

Currently, ocular melanoma is the second most common type of melanoma after cutaneous melanoma and comprises 3–4% of all melanomas in the United States followed by mucosal melanoma [3,4]. Ocular melanoma can be divided into uveal and nonuveal ocular melanoma. Uveal melanoma (UM) is the largest group of ocular melanoma and consist of choroid, ciliary body, and iris melanoma. Nonuveal melanomas are all conjunctival melanomas (CM) [3]. In almost all cases, one eye is affected because bilateral ocular melanoma is only reported in 0.1% [4,5]. Mean age of diagnosis in UM and CM is comparable (61.4 and 61.7 years old, respectively) in Caucasians [4], although the age of onset of UM is lower in Asian patients [6]. The overall cancer-specific relative survival in UM is slightly higher compared to CM whereas the mean cancer-specific survival at 5 years is equal [4].

### 1.1. Uveal Melanoma

**Figure 1:**
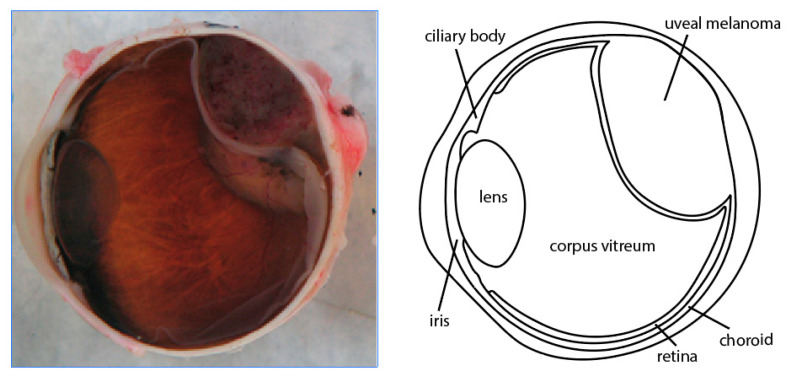
Section of an eye with a uveal melanoma in the choroid (**left**), schematic overview of the anatomy (**right**).

**Figure 2:**
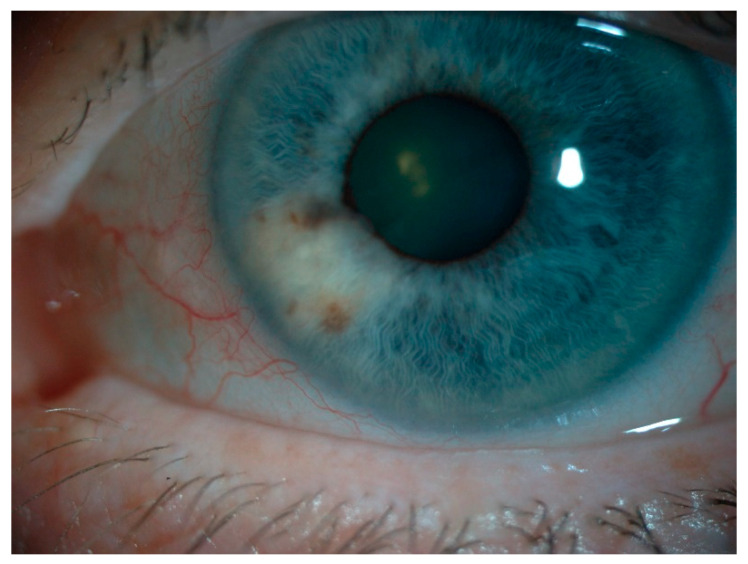
Partly amelanotic iris melanoma.

UM is the most common primary intraocular malignancy in adults in the Western world with an incidence of 5–7:1,000,000 people [4,5]. UM arises predominantly in the choroid followed by ciliary body and iris (Figure 1) [7,8]. The prognosis of iris melanoma is favorable compared to melanoma of the choroid and ciliary body [9,10]. The 5-year overall survival of choroid and ciliary melanoma is 77–80% with a cancer specific 5-year survival rate of 76%. More than half of all patients develop metastases with a median survival of six months when metastatic disease is present in UM, whereas the melanoma related death in iris melanoma is 3–4% [4,11,12,13]. One study even showed a 15-year melanoma specific survival up to 100%. [10] Although both groups have resemblances in their genetic makeup, the involvement of certain genes is different as well as their clinical behavior. Therefore, iris melanomas are considered a distinct subgroup within UM. UM used in the literature generally refers to posterior (choroid and ciliary body) UM. UM can appear as (partly) amelanotic lesions as shown in Figure 2.

Treatment of primary UM consist of surgery, radiotherapy or a combination of these therapies [4]. A large randomized trial has shown that treatment modality has no effect on long-term survival [14]. However, in small UM, radiotherapy might have a beneficial effect over surgery regarding overall survival, although high-risk patients were not identified within this study [15]. Unfortunately, there is no adequate treatment available for metastatic UM. First line treatment with immunotherapy, using an anti-PD-1 monoclonal antibody named pembrolizumab, showed only positive results in a minority of patients [16]. Since *GNAQ* and *GNA11* genes, in which mutations occur in UM [17], are related to the MAPK-Erk Pathway, inhibitors of this pathway (MEK inhibitors) could have an effect of metastatic disease. However, clinical trials using MEK inhibitors in metastatic UM show contradictory results [18,19].

Mutations in certain genes as well as chromosomal aberrations correlate with patient prognosis [20,21]. Not only are genetic and cytogenetic characteristics correlated to prognosis, but several clinical and histopathological characteristics are also associated with a higher risk of metastatic disease. Clinical and histopathological parameters correlated with a poor prognosis are larger tumor diameter, ciliary body involvement, mixed or epithelioid cell type, extracellular matrix patterns and high mitotic or Ki-67 proliferation index [22].

### 1.2. Pediatric Uveal Melanoma

UM in children and young adults are described in less than 1% of all UM [8,23,24,25]. Like in adult UM, the tumor is most commonly primarily located in the choroid. Contrary to the frequency in adults, however, the frequency of iris melanoma is higher than melanoma originating from the ciliary body. A large cohort of 8033 UM patients described by Shields et al. showed in the age group of patients of 20 years or younger that 21% of developed UM are iris melanoma, whereas in adults, only 2–4% of UM consists of iris melanoma [8]. Other studies describe iris melanoma in about 20–25% of all UM patients in children and young adults [24,26]. It seems that females are at more risk to develop UM before the age of 25, although this was not statistically significant in cohort studies, probably due to the small size of the groups [26,27,28].

Treatment of pediatric UM does not differ from the treatment in adults and includes enucleation, radiotherapy, resection and proton beam therapy [24,25,27]. Few children are treated with laser photocoagulation, transpupillary thermotherapy, gamma knife and photodynamic therapy [26,27].

The prognosis of UM in children and young adults differs from adult patients. Children with UM have a favorable prognosis compared to young adults with UM (18 to 24 of age) with a 10-year survival rate of 92% and 80%, respectively [27]. With a 10-year survival rate of 93%, juvenile UM patients have a better prognosis compared to adults (65%) [24]. Metastasis of UM (posterior and iris melanoma) in patients with age at diagnosis <21 years are described in 8–44% [8,24,25,29,30] with congenital melanocytosis as a predictor of poor prognosis [27]. Patients with extraocular extension also have a significant higher risk of UM-related death, whereas ciliary body involvement or cell type had no effect on prognosis [27]. Although not statistically proven, it seems that females tended to have a worse survival compared to males [27,28].

### 1.3. Conjunctival Melanoma

**Figure 3:**
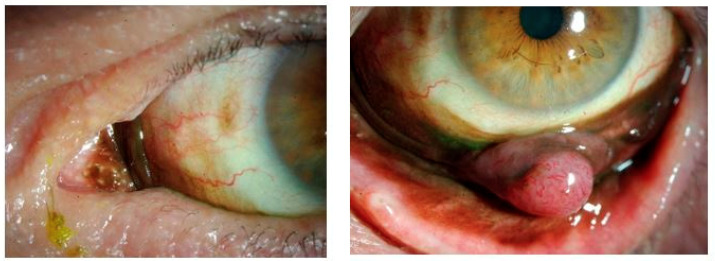
Primary acquired melanosis (**left**), conjunctival melanoma (**right**) in the same patient.

Conjunctival melanoma (CM) arises from melanocytes in the epithelium of the conjunctival membrane and account for less than 10% of all ocular melanoma [4,5,9]. The incidence of CM is 1–2 per 1,000,000 people in the Western world with an increasing trend [31,32]. Malignant lesions account for 6–30% of all conjunctival lesions, with squamous cell carcinoma and melanoma being the most frequent [31,33]. CM most often arise from primary acquired melanosis (PAM) but CM originating from nevi or de novo also occurs (Figure 3) [34,35]. Conjunctival melanoma in children is rare, a systematic review of the literature from 2019 by Balzer et al. described 32 patients with conjunctival melanoma with an age of onset before 18 years [36].

Treatment consist of excision in most cases, preferably in combined with cryotherapy [4,33]. Cryotherapy as single treatment is uncommon as well as enucleation Other less used treatments are topical or injection chemotherapy, exenteration, plaque radiotherapy, external beam radiotherapy and systemic chemotherapy [33].

Risk factors for metastasis (nodal and distance) are a higher tumor thickness, histologic ulceration and the presence of mitotic figures [37]. 12–26% of patients develop metastasis within 5 years after diagnosis [38,39]. Distant metastases were found in the liver, lung, brain or elsewhere. Local recurrence occurs in almost one third of patients [38]. Like in uveal melanoma, no standardized therapy is available for disseminated disease. However, small case series show the potential use of target therapy and immunotherapy in advanced local and metastatic conjunctival melanoma [40,41].

## 2. Genetics of Ocular Melanoma

Chromosomal aberrations are a key feature of genomic instability of cancer cells and the observation of chromosome 3 loss in metastasizing UM by Prescher et al. in 1996 was a cytogenetic hallmark for UM [42]. With the introduction of new high throughput DNA sequencing techniques, replacing traditional karyotyping, a number of genes mutated by the different types of ocular melanoma were discovered. Their role in initiation or progression of the disease was investigated and will be discussed in the next sections for some of these genes.

### 2.1. Uveal Melanoma

The most frequently mutated genes in UM are *GNAQ* and *GNA11*. Mutations in these genes occur in 71–93% of all UM tumors; we and others have shown that they have no predictive value [22,43,44,45]. Mutations occur most often in the 209 residue of exon 5 although mutations in amino acid 183 in exon 4 are also described [45]. *GNAQ* and *GNA11* are involved in the Gα signaling pathway and are mutually exclusive in the vast majority of tumors. Other frequently mutated genes in UM are *BAP1*, *SF3B1* and *EIF1AX* [21,46].

Prognosis of UM patients can be predicted with the mutation status of secondary driver genes *EIF1AX*, *SF3B1* and *BAP1* which are almost always mutually exclusive. Patients with a *BAP1* mutation or absent BAP1 expression with immunohistochemistry (IHC) have a high metastatic risk while patients with an *EIF1AX* mutation have a low risk of metastatic disease [20,21,47]. Mutations in *BAP1* are less frequently found in iris melanoma and not correlated with survival [44]. When looked at the chromosomal profile of UM, there is a decreased disease-free survival in tumors with loss of chromosome 3 (monosomy 3) which is associated with *BAP1* mutations [20,22,47]. Gain of chromosome 8q is a poor predictor as well [48]. Other chromosomal aberrations found in UM include chromosome 1p loss and gain of chromosome 6 [20,22]. These chromosomal aberrations can be detected using karyotypes, fluorescent in situ hybridization (FISH) and single nucleotide polymorphism (SNP) array analysis.

In patients harboring a disomy 3 UM, two genes are mainly mutated: *EIF1AX* and *SF3B1.*

In eukaryotes, *EIF1AX*, encoding for the X-linked Eukaryotic Translation Initiation Factor 1A protein, stimulates and stabilizes the ribosome and is involved in start codon recognition [49,50,51,52]. In UM, mutations in *EIF1AX* primarily occur as heterozygous amino acid substitutions in exon 1 and 2, causing an in-frame mutation affecting the proteins N-terminus [53,54]. As EIF1AX acts as a regulator for translation initiation, mutations herein result in wrong selection of start sites, which might cause suppressed translation of canonical transcripts or upregulation of oncogenes [53,55]. However, the precise biological function and its contribution to tumorigenesis is not fully understood. Although the heterozygous mutation is located on the X-chromosome, in females, the wild-type allele is silenced through selective X-chromosome inactivation, resulting in mutant transcripts only [53].

Yavuzyigitoglu et al. have shown that patients harboring an *EIF1AX* tumor have a good prognosis, as these tumors harbor a low risk of metastasis [23]. *EIF1AX* mutations are reported to occur in 8% to 19% [56].

*SF3B1* encodes for a part of the spliceosome, splicing factor 3 subunit 1. The spliceosome is responsible for splicing noncoding introns from precursor mRNA at specific splice sites, leaving only the exonic sequence [57]. As part of the spliceosome, the SF3b complex recognizes branch point sites on precursor mRNA at which U2 snRNP (small nuclear ribonucleoprotein) is recruited. SF3b facilitates the interaction between the branch point site and U2 snRNP by protein crosslinking, after which the spliceosome is catalyzed [58,59,60]. The majority of recurrent hotspot mutations in SF3B1 occur at the edge of the C-terminal HEAT domains, near the precursor mRNA binding region and might be important for RNA or protein interactions [57,61].

Mutations in *SF3B1* lead to aberrant transcripts [58], primarily caused by alternative 3′ splice site selection upstream of the canonical splice site, coincided by misregulated branch point usage [62]. As a result, mutations in spliceosome components, such as *SF3B1* mutant tumors, can have alternative 3′ acceptor splice sites, alternative cassette exons and intron retention in protein coding and noncoding genes as shown by Furney et al. [63].

Yavuzyigitoglu et al. reported UM patients harboring an *SF3B1* mutation were diagnosed younger at 54.5 years than patients harboring an EIF1AX or BAP1 mutated tumor, diagnosed at 64 years [21]. They reported that these patients have an apparent risk of late onset metastases as 11 of 32 (34%) metastasized within 16 years (mean: 11.2 years after initial diagnosis) [23]. Harbour et al. described that 18.6% of the UM mutations occur in *SF3B1* [64].

Because of the small number of iris melanoma, the genetic background is not as extensively explored as posterior uveal melanoma. Although genes involved in posterior uveal melanoma are mutated in iris melanoma as well, there are some differences. Iris melanoma harbor *GNAQ*, *GNA11* and *EIF1AX* mutations while *BAP1* mutations and mutations in *SF3B1* are less common or rare [43,44]. A mutation in *BRAF*, a gene often mutated in cutaneous melanoma, was identified in iris melanoma [44]. Loss of chromosome 3 is described in iris melanoma as well as loss of 9p [65]. Moreover, aberrations of chromosome 1, 6 and 8, chromosomes that are involved in posterior uveal melanoma as well, were described in iris melanoma [66,67].

More sequencing and larger UM patient cohorts identified less prevalent recurring genes. Mutations in *PLCB4*, a downstream effector of Gαq signaling are described in <10% of uveal melanoma [68]. A study aimed at identifying gene mutations in 139 UM showed mutations in *GNAQ* and *GNA11* in 93% being mutually exclusive except for one UM harboring a *GNAQ* and *GNA11* mutation. Mutations in *PLCB4* (2%) were found in tumors with or without a *GNA11* mutation, whereas mutations in *CYSLTR2* (5%) were identified in UM with no mutation in one of the other genes. Deletions in spliceosome factors *RBM10*, in-frame deletions of *SRSF2* and homozygous deletion *SF3A1* were found in only a few tumors [69]. Mutations in *SRSF2* were all heterozygous in-frame deletions and starting at residue 92 or 03, except for one case described in The Cancer Genome Atlas (TCGA) starting at 174 [70].

### 2.2. Conjunctival Melanoma

Mutations in *BRAF* are identified in 25–35% of conjunctival melanoma of which the vast majority is the *BRAF* V600E mutation. This mutation can be identified using genetic testing, but immunohistochemistry is used as well [34]. The *BRAF* gene is involved in signal transduction and mutated in different types of cancer, most commonly in malignant melanoma. Amino acid valine (V) at residue 600 is mutated and replaced by a glutamic acid (E) in cutaneous melanoma [71]. BRAF mutations were more often identified in conjunctival melanoma with a bulbar localization [34]. Apparently, cutaneous melanoma and conjunctival melanoma have an overlap in their genetic background. Other genes involved in the development of conjunctival melanoma are *TERT* promoter, *NRAS* and *NF1* in which pathogenic mutations are described [35,72,73]. *GNAQ* and *GNA11* mutations are identified, but not the activating hotspot mutations that occur in UM [35]. Amplification of chromosome 6 is found in more than half of the conjunctival melanoma. Moreover, alterations in chromosome 9q, 11q, 6p, 17p and 19 have also been detected [39]. TERT promoter mutations have recently been identified to correlate to metastatic disease [74].

## 3. Inheritance of Uveal Melanoma

**Figure 4:**
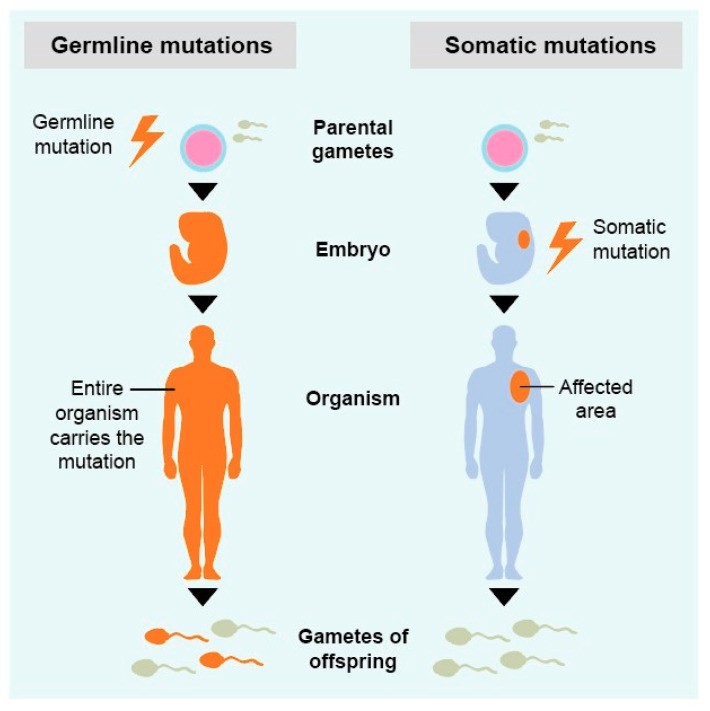
Schematic overview of germline and somatic mutations.

Mutations or variants in genetic information can be passed from one generation to the next (inheritance) and cause a specific phenotype or disease. This is only possible if the mutation is present in the gametes, which is in general a germline mutation (Figure 4). Somatic mutations occur during embryogenesis or throughout life and are not present in the gametes and therefore not heritable.

Only 2–4% of all uveal melanoma patients harbor a germline *BAP1* mutation [75,76,77] and although familial uveal melanoma is rare, *BAP1* has been identified as a predisposition gene for UM as well as a variety of other cancers [78]. When focused on familial UM, the incidence of *BAP1* germline mutations is higher and is reported up to 19%. Not only UM was described in these families but other cancers such as cutaneous melanoma and renal cell carcinoma were present in family members with this *BAP1* tumor predisposition syndrome (BAP1-TPDS) [79]. Almost all UM in patients with a germline *BAP1* mutation have tumors that are located posteriorly, although one iris melanoma has been described [80]. In general, more cutaneous melanoma and ocular melanoma in the family history was reported in patients with uveal melanoma and a *BAP1* germline mutation compared to patients without this germline mutation. Moreover, germline mutated *BAP1* carriers have a larger tumor diameter and more frequently reported ciliary body involvement. Multivariate analysis did not show that germline *BAP1* mutations are an independent risk factor for the development of metastasis [75]. When metastasis-free survival of UM patients with a germline *BAP1* mutation was compared to those with a somatic *BAP1* mutation, it was shown that the germline *BAP1* mutated group has a more favorable prognosis [81]. In contrast, another study showed that germline *BAP1* mutations occur more often in metastatic ocular melanoma compared to nonmetastatic ocular melanoma, even though this difference was not significant and not adjusted for the greater risk of metastatic disease in *BAP1*-mutated UM in general [82]. The median age of diagnosis does not differ between patients with a somatic or germline BAP1 mutation [81].

The four main tumor types strongly associated with the BAP1-tumor predisposition syndrome (*BAP1*-TPDS) are uveal melanoma, mesothelioma, cutaneous melanoma and renal cell carcinoma [83]. The frequency of *BAP1* germline mutations is higher in families with cutaneous and uveal melanoma compared to families without uveal melanoma [82]. In families with a positive family history of UM, the frequency of *BAP1* germline mutations was 22% [77]. Accordingly, an accurate family history should be obtained when diagnosing new UM. In addition, it has been shown that germline null mutations in *BAP1* are more frequently observed compared to controls and the *BAP1*-TPDS is probably underreported [84]. Therefore, *BAP1* germline testing might be useful in case of familial UM or the occurrence of other cancers in a patient’s family history. Other germline mutations described in UM are mutations in the TP53 gene, although these are rare and the role of these mutations should be elucidated [85] TP53 mutations associated with UM and breast cancer in a family are already described in 1905. This was probably in the context of the Li–Fraumeni syndrome [86].

## 4. Prognosis

### 4.1. Uveal Melanoma

Several clinical and histopathological characteristics of UM are used to predict patients’ prognosis. Initially, it was found that histopathologic features as cell type, largest tumor diameter and the location of anterior margin were correlated to different risk class of melanoma-related survival [87]. Other predictors for poor outcome were scleral extension, mixed/epithelioid cell type, Ki-67 proliferation index, inflammatory phenotype, high mitotic figures and deeper scleral extension and the presence of extracellular matrix patterns [22,87,88]. However, some of these characteristics are not independent of each other. For example, larger tumors are more commonly found in the anterior choroid or ciliary body and feature epithelioid cells [89].

Not only can clinical and histopathological characteristics of the tumor be used to predict patients’ prognoses, but patient characteristics are also important factors. Patients who develop UM before the age of 21 have a better prognosis compared to middle-aged adults (until age 60) or older patients [8,30]. It should, however, be mentioned that tumor thickness and diameter was not equally distributed between all age groups [8]. Besides age at diagnosis, there are studies showing male patients are at higher risk for the development of metastases than female patients [90,91]. Moreover, metastatic disease developed earlier in men and the survival rate from diagnosis of metastatic disease was lower [90]. This difference in sex as a risk factor was not detected in Asian populations and the metastasis-free survival was higher compared to previous mentioned studies [6]. Uveal melanoma does occur in pregnancy, although the survival rates are similar to non-pregnant women [92].

**Figure 5:**
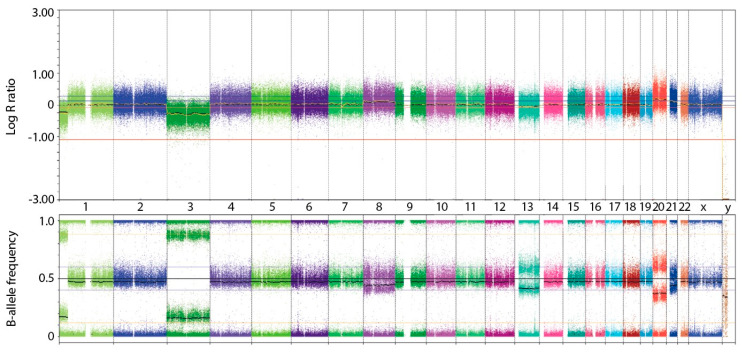
SNP-array profile of a uveal melanoma with partial loss of chromosome 1p and loss of chromosome 3. The *x*-axis represents the chromosomes. The upper figure displays the Log R ratio; in the lower figure, the B-allele frequency is showed.

Later on, genetic factors such as chromosomal aberrations and genetic mutations were added to improve prediction of patients’ survival. It was shown that patients with UM and loss of chromosome 3 (monosomy 3) [93,94,95] and gain of chromosome 8q in the tumor had a significant poor prognostic influence [22,94,95]. In addition, monosomy 3 was an independent risk factor for the development of metastasis, and thus poor prognosis, when corrected for tumor diameter and tumor site. Nevertheless, this study did not show a correlation of histological cell type, extrascleral extension and tumor thickness to prognosis [42]. One of the methods used in cytogenetics to detect chromosomal aberrations is FISH. This technique is used in UM to confirm the use of chromosome 3 and 8 and their relation to prognosis [96]. Further research validates the fact that patients with a UM showing monosomy 3 have a significantly lower disease-free survival. In addition, a relation between concurrent loss of 1p and 3 and the risk of metastasis was shown. UM that harbor both chromosomal aberrations is at an even higher risk of developing metastasis than UM with solely loss of chromosome 3. There was also a relation between cell type and the existence of chromosome 3 loss or 6p gain [93]. Loss of 1p and 8 are significant prognostic factors independently [91]. In order to display these chromosomal aberrations, SNP-array analysis can be used. SNP-array analysis is a technique which is frequently used in UM research (Figure 5).

For example, greater tumor thickness or larger diameter correlates with partial or complete monosomy 3 [97]. This implies that the histopathological risk factors previously described are not independent of the genetic background of the primary tumor. This study also showed that the patients with UM harboring partial monosomy have better prognoses compared to those with complete monosomy 3 [97], although later studies showed no significant difference in survival between patients with monosomy 3 or partial loss of chromosome 3 of the primary UM. In addition to these findings, loss of heterozygosity of chromosome 3 is even more important than monosomy 3 by itself [98].

**Figure 6:**
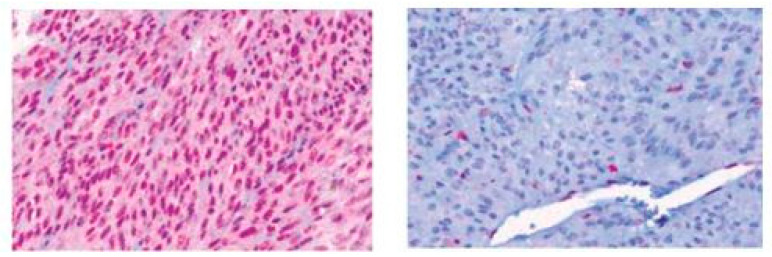
Microscopic overview of uveal melanoma with BAP1 expression 400× (**left**) and absence of BAP1 expression 400× (**right**).

The role of BRCA1-associated protein (BAP1), located on chromosome 3, was proposed to have a role on the prognosis of UM about a decade ago. It was found that somatic mutations in *BAP1* were frequently present in metastasizing UM [99]. Immunohistochemistry (IHC) can be used to detect the presence of BAP1 protein expression (Figure 6). Nuclear BAP1 expression is strongly correlated with patient survival and metastatic rate; lack of expression is a risk factor for the development of metastasis and poor prognosis [100,101]. It was shown that *BAP1* mutations often results in the absence of BAP1 expression using IHC. Moreover, there is an association of BAP1 loss and monosomy 3 of the primary tumor [102].

When looking at specific gene mutations, there are several other genes described which can be used for prognostication besides *BAP1*. It has been shown that patients with UM harboring an *EIF1AX* mutation have prolonged survival and low risk of metastasis [6,21]. These somatic mutations mainly occur in UM with disomy 3 [21,46,55]. Another mutation frequently found in disomy 3 UM is a hotspot missense mutation in *SF3B1* at codon 625 [21,55]. Mutations in this gene, encoding subunit 1 of splicing factor 3b, are almost in all cases affecting codon 625, but mutations in K666 or K700 are also described [53,54,63,64,65,66,67,68,69,70,71,72,73,74,75,76,77,78,79,80,81,82,83,84,85,86,87,88,89,90,91,92,93,94,95,96,97,98,99,100,101,102,103,104,105,106,107,108]. Mutations in *SF3B1* are associated with alternative splicing of a wide range of target genes [63]. These findings were also identified in RNA sequencing data.

The clinical relevance of these splicing events is not completely clear, but it has been shown that *SF3B1* mutated tumors are at risk to metastasize. Patients with UM harboring an *SF3B1* mutation can develop late onset metastases. Metastases develop in most patients after 5 years, and metastatic disease can occur even after 10 years. This is in contrast with BAP1-mutated UM, in which metastases are mainly diagnosed within 5 years after diagnosis [21].

Not only have chromosomal aberrations and mutation status of the tumor have been used to classify uveal melanoma patients, but a classification can also be performed with gene expression profiling. Two profiles can be distinguished, with class 1 being tumors with a good overall survival and low metastatic risk, whereas class 2 tumors are more likely to metastasize [103]. The ability to differentiate two groups of UM based on gene expression profiling correlating with survival was also shown in other studies [104,105]. This subgroups classification is not only based on gene expression profiling but corresponds with mutational status and micro-RNA expression as well. These different mi-RNA expression profiles are probably not caused by the copy number state of the primary tumor but act as an independent process [106]. This mi-RNA expression profile can contribute to the prediction of patient prognosis. When looked to overall survival, the upregulation or downregulation of certain mi-RNAs have a prognostic value in patients with UM [107].

### 4.2. Iris Melanoma

The prognosis of iris melanoma is favorable compared to posterior uveal melanoma. A large cohort of more than 1000 iris melanoma showed that 3% of iris melanoma metastasized [109]. This finding is according to smaller cohort studies in which metastatic disease is present in 10% or less [11,44,110,111].

The American Joint Cancer Committee (AJCC) on Cancer classification can be used to describe and predict patient outcome. Most patients (75%) are scored following the AJCC Classification eighth edition as T1 (limited to the iris), whereas tumor confluent with or extending into the ciliary body and/or choroid (T2) including scleral extension (T3) or extra scleral extension (T4) are less common [11]. The 10-year risk of metastatic disease has been shown to be 5% in T1 tumors. Iris melanomas that are classified as T4 showed a 33% estimate of metastasis at 5 years, although only 5% of all iris melanomas were T4 tumors in this study [11]. Extraocular extension and high intraocular pressure are described as risk factors for metastasis [110]. Histological cell type is a risk factor as well; mortality was lower in spindle cell melanoma compared to mixed and epithelioid cell melanoma [108,111]. Within different age groups, there is no significant difference in survival between children, middle-aged adults and older adults [110]. BAP1 status using immunohistochemistry was not found of predictive value [44].

Recurrent disease was higher in patients treated with iodine-125 radioactive plaque therapy in which there was reduced cornea surface coverage by the plaque and the presence of glaucoma after treatment. These risk factors were not correlated with the metastatic rate [112].

Regarding the good prognosis after treatment, it should be noted that overtreatment could be possible in patients with iris melanoma. A large cohort of suspicious melanocytic iris lesions showed the low potential for malignant transformation and good prognosis [113]. This indicates that an overestimation of favorable prognosis after treatment is possible in patients who could have underwent conservative treatment as well.

### 4.3. Conjunctival Melanoma

Local recurrence rates of conjunctival melanoma are described in 30%–58% [40,114]. Treatment with excision alone has a higher risk of recurrent disease [40,114] as well as nonepibulbar location of the tumor [114,115]. The 5-year overall survival is 72% (melanoma-related survival 90%) [40]. Metastasis are reported in literature in about a quarter of conjunctival melanoma patients [39,116].

Metastasis of conjunctival melanoma occur to regional lymph nodes, but distant metastases are described as well [39,40]. Distant metastases are found in patients following lymph node involvement, but are also described in patients without lymph node metastasis [40]. A correlation between tumor thickness (>2 mm), ulceration and mitotic figure count (>1/mm^2^) and regional lymph node metastasis was found [37,74,117] Tumor diameter was also correlated with the risk of regional metastasis in a Dutch cohort [116]. Cell type is an important risk factor since patients with mixed cell type tumors had a higher mortality compared to spindle cell CM [109]. When lymphangiogenesis is present, a higher recurrence and risk of metastatic disease is present [109,118]. However this might be a confounder since high lymphatic density was associated with risk factors that are described as independent factors previously such as greater tumor thickness and larger tumor diameter [118]. In patients who underwent sentinel lymph node biopsy, a positive biopsy was related with a higher incidence of distant metastasis and a worse disease specific survival [37], and local recurrence is associated with a higher risk of melanoma-related death [40]. Similar findings were reported using a large Chinese cohort: a higher T stage using the AJCC staging system, greater tumor thickness, more quadrants involved, local resection and the absence of adjuvant therapy were associated with worse survival [119].

Chromosomal status is also correlated with survival, and it has been shown that deletions on chromosomal 10q are correlated with metastatic disease [39].

*BRAF* mutations occur frequently in conjunctival melanoma, especially in the sun-exposed area of the bulbar conjunctiva. However, no association of survival and gene mutation status regarding *BRAF* and *KIT* was identified [119]. This was confirmed in another study in which no relation between *BRAF* mutation status and local recurrence, metastasis and death is observed [34].

The presence of *BRAF* mutations might be important in the future because BRAF/MEK inhibitors could possibly play a role in treatment of metastatic disease, as they is used in cutaneous melanoma where *BRAF* mutations at the same residue are present [120]. *TERT* promotor mutations correlate with prognosis which could act as a therapeutic strategy in the future [74]. The survival in children appears favorable compared to adults [36]. However, the incidence in children and adolescents is low and the groups described in literature small.

## 5. Noninvasive Testing

Tumor tissue is needed to predict patients’ prognoses; prognostication of patients who undergo eye preserving treatment such as radiotherapy is not possible based on a genetic profile when no biopsy was taken. Biopsies of tumor tissue are invasive with an inherent risk. Therefore, there is a need for noninvasive tumor testing which can not only be used for diagnostic purposes, but also to monitor the disease with a biomarker in real time. For other cancer types, noninvasive testing is widely used for diagnostics and follow-up of patients and includes cell free DNA (cfDNA), circulating tumor DNA (ctDNA), circulating tumor cells (CTC), tumor-derived exosomes, tumor-educated platelets and micro-RNA. These so-called liquid biopsies can be withdrawn from plasma, urine and other body fluids. One of the advantages of liquid biopsies over tissue biopsy is that tumor heterogeneity is more represented and changes of the mutational landscape of the tumor over time could be detected.

In lung and breast cancer, cfDNA concentrations correlate with disease progression [114,121]. Moreover, tumor-specific mutations could be detected in cfDNA from plasma, indicating that this technique can be used as a diagnostic as well as predictive tool [115,122]. Methods to isolate CTCs are well investigated and it has been shown that the prevalence of CTCs in blood in patients of several metastatic cancer types was risen [123].

The use of CTCs in UM seems to have a predicted value on overall survival, although only small studies have been performed. Patients with CTCs detected in early-stage uveal melanoma have a less favorable prognosis compared to patients in which CTCs were not detected [124]. In patients with metastatic UM, the CTC cell count and ctDNA levels were also correlated with progressive free survival. There was also a relation with clinical characteristics of the tumor and the level of CTCs and ctDNA. More CTCs and higher levels of ctDNA in the blood were detected when the tumor volume was higher. In case of miliary hepatic metastases, resembling many small diffuse metastases in the liver, the ctDNA and CTC count was higher. This correlation was not found in patients with extrahepatic metastases [125]. When looked at chromosomal aberrations in the primary tumor, the CTC and cfDNA cells show an overlapping genetic profile. Moreover, *GNAQ* and *GNA11* mutations were detected in ctDNA of UM patients. *CYSLTR2* and *PLCB4* mutations were detected in only two patients. The detection rate of ctDNA was much lower in patients with localized UM (27%, *n* = 30) compared to patients with metastatic disease (100%, *n* = 7) [126].

When looked at mRNA expression in the blood of patients with UM using reverse transcription PCR, it has been shown that the detection of CTCs with this method can be used for prognostication as well. mRNA expression of tyrosinase and MelanA/MART1 were correlated with disease-specific survival and overall survival [127]. Moreover, tyrosinase expression is significantly different when the primary tumor was classified regarding the tumor size. Tyrosine expression was the highest in large tumors, and there was a direct correlation between CTC values and tyrosine levels. The overall survival and disease-free survival were also better in patients without tyrosinase expression in their blood [128].

Another entity that can be used for noninvasive testing in patients with cancer are exosomes. Exosomes are nanosized extracellular vesicles containing proteins, RNA and DNA excreted by cells and have functional properties [129]. Although UM is a relatively small tumor, the concentration of circulating exosomes derived from plasma of patients with metastatic uveal melanoma is higher compared to healthy controls. These exosomes contain melan-A and melanoma-associated micro-RNAS which support the theory that these exosomes are of metastatic melanoma origin [130]. These findings emphasize the fact that there is an enrichment of exosomes derived from cancer cells.

These methods give promising results of new techniques that can be used in the prognostication of patients with UM in a noninvasive manner. However, challenges will be faced due to tumor size and the heterogeneity in affected genes, especially the nonhotspot mutations that occur in *BAP1*.

## 6. Conclusions

Although posterior uveal melanoma, iris melanoma and conjunctival melanoma are all ocular melanoma, they are distinct subtypes. The genetic profile of the type of ocular melanoma differs from one to the other. Mutations that are common in posterior UM such as *GNAQ* and *GNA11* are described in iris melanoma but in lower frequency [44]. Moreover, mutations in *BRAF* were detected in only one iris melanoma [44], whereas *BRAF* mutations are common in conjunctival melanoma [34]. Germline mutations in *BAP1* are described in UM, but conjunctival melanoma is not part of the *BAP1* tumor predisposition syndrome. In familial UM as well as families in which UM is present and family members known with malignant mesothelioma, cutaneous melanoma, clear cell renal cell carcinoma and basal cell carcinoma, an underlying germline mutation in *BAP1*, can be present. Therefore, it is recommended to test for germline *BAP1* mutations when the family history is suspect.

Not only the genetic background of these melanomas is different. The overall survival of patients with ocular melanoma of the different subtypes differs broadly. The prognosis of posterior UM is poor compared to iris melanoma and the metastatic site differs between UM and conjunctival melanoma. In conjunctival melanoma, metastases to regional lymph nodes are described frequently, whereas UM primarily metastasize to the liver. The underlying pathogenesis of difference in survival is not yet clarified. It has been shown that *BAP1* mutations are correlated with poor prognosis in UM patients [100]. Since about half of all patients with posterior UM harbor a mutation in the *BAP1* gene, prognosis is poor. *SF3B1* mutations, correlated with late onset metastases, have been found more often in posterior UM compared to iris melanoma [21,44]. Based on the genetic profile, the difference in survival could be explained.

The time of diagnosis of iris melanoma is probably in an earlier stage compared to posterior UM since patients can detect changes in the iris. Posterior UM, in contrast, can be detected without any clinical symptoms by routine clinical examination. Loss of vision can occur when the melanoma is present in the macular region or when retinal detachment is present. It seems that UM with retinal detachment at presentation carries a higher risk of metastases. However, this risk can be attributed to the larger tumor diameter and other tumor characteristics [131]. Retinal detachment is therefore not a risk factor on its own. It is possible that these tumors are more aggressive, and therefore are detected at a larger tumor size. However, the time of diagnosis is probably earlier compared to tumors that do not give rise to any clinical symptoms. Therefore, it cannot be stated that iris melanoma has a better prognosis compared to posterior UM due to a probably earlier time of diagnosis.

Conjunctival melanoma and UM are both rare in children; however, the limited data suggest that survival in children seems better in both groups.

Unfortunately, no current treatment is available for metastatic disease of ocular melanoma. In UM, liver resection is possible in only a few cases, but no validated systemic treatment is currently used. Targeted treatment for conjunctival melanoma harboring a *BRAF* mutation could be considered since the genetic profile is similar to that of cutaneous melanoma in which BRAF/MEK inhibitors are used. Further genetic and molecular testing is needed to gain more insight in ocular melanoma and hopefully lead to targets for therapeutic use.

## Data Availability

No new data were created or analyzed in this study. Data sharing is not applicable to this article.

## References

[1] Kivela T.T. (2018). The first description of the complete natural history of uveal melanoma by two Scottish surgeons, Allan Burns and James Wardrop. Acta Ophthalmol..

[2] Zozolou M., Tsoucalas G., Karamanou M., Laios K., Georgalas I., Douzenis A., Androutsos G. (2018). The distinguished Austrian ophthalmologist Ernst Fuchs (1851–1930) and the “sarcom des uvealtractus”. J. BUON.

[3] McLaughlin C.C., Wu X.C., Jemal A., Martin H.J., Roche L.M., Chen V.W. (2005). Incidence of noncutaneous melanomas in the U.S. Cancer.

[4] Mahendraraj K., Shrestha S., Lau C.S., Chamberlain R.S. (2017). Ocular melanoma-when you have seen one, you have not seen them all: A clinical outcome study from the Surveillance, Epidemiology and End Results (SEER) database (1973–2012). Clin. Ophthalmol..

[5] Isager P., Osterlind A., Engholm G., Heegaard S., Lindegaard J., Overgaard J., Storm H.H. (2005). Uveal and conjunctival malignant melanoma in Denmark, 1943–1997: Incidence and validation study. Ophthalmic Epidemiol..

[6] Yue H., Qian J., Yuan Y., Zhang R., Bi Y., Meng F., Xuan Y. (2017). Clinicopathological Characteristics and Prognosis for Survival after Enucleation of Uveal Melanoma in Chinese Patients: Long-term Follow-up. Curr. Eye Res..

[7] Mahendraraj K., Lau C.S., Lee I., Chamberlain R.S. (2016). Trends in incidence, survival, and management of uveal melanoma: A population-based study of 7516 patients from the Surveillance, Epidemiology, and End Results database (1973–2012). Clin. Ophthalmol..

[8] Shields C.L., Kaliki S., Furuta M., Mashayekhi A., Shields J.A. (2012). Clinical spectrum and prognosis of uveal melanoma based on age at presentation in 8033 cases. Retina.

[9] Isager P., Engholm G., Overgaard J., Storm H. (2006). Uveal and conjunctival malignant melanoma in denmark 1943-97: Observed and relative survival of patients followed through 2002. Ophthalmic Epidemiol..

[10] Jamison A., Bhatti L.A., Sobti M.M., Chadha V., Cauchi P., Kemp E.G. (2019). Uveal melanoma-associated survival in Scotland. Eye (London).

[11] Shields C.L., Di Nicola M., Bekerman V.P., Kaliki S., Alarcon C., Fulco E., Shields J.A. (2018). Iris Melanoma Outcomes Based on the American Joint Committee on Cancer Classification (Eighth Edition) in 432 Patients. Ophthalmology.

[12] Lane A.M., Kim I.K., Gragoudas E.S. (2018). Survival Rates in Patients After Treatment for Metastasis From Uveal Melanoma. JAMA Ophthalmol..

[13] Rao Y.J., Sein J., Badiyan S., Schwarz J.K., DeWees T., Grigsby P., Rao P.K. (2017). Patterns of care and survival outcomes after treatment for uveal melanoma in the post-coms era (2004–2013): A surveillance, epidemiology, and end results analysis. J. Contemp. Brachyther..

[14] Collaborative Ocular Melanoma Study G. (2006). The COMS randomized trial of iodine 125 brachytherapy for choroidal melanoma: V. Twelve-year mortality rates and prognostic factors: COMS report No. 28. Arch. Ophthalmol..

[15] Jang B.S., Chang J.H., Oh S., Lim Y.J., Kim I.H. (2017). Surgery vs. radiotherapy in patients with uveal melanoma: Analysis of the SEER database using propensity score matching and weighting Chirurgie vs. Strahlentherapie bei Patienten mit Uveamelanom: Analyse der SEER-Datenbank mithilfe von Propensity-Score-Matching und -Gewichtung. Strahlenther. Onkol..

[16] Rossi E., Pagliara M.M., Orteschi D., Dosa T., Sammarco M.G., Caputo C.G., Petrone G., Rindi G., Zollino M., Blasi M.A. (2019). Pembrolizumab as first-line treatment for metastatic uveal melanoma. Cancer Immunol. Immunother..

[17] Van Raamsdonk C.D., Griewank K.G., Crosby M.B., Garrido M.C., Vemula S., Wiesner T., Obenauf A.C., Wackernagel W., Green G., Bouvier N. (2010). Mutations in GNA11 in uveal melanoma. N. Engl. J. Med..

[18] Luke J.J., Olson D.J., Allred J.B., Strand C.A., Bao R., Zha Y., Carll T., Labadie B.W., Bastos B.R., Butler M.O. (2019). Randomized phase II trial and tumor mutational spectrum analysis from cabozantinib versus chemotherapy in metastatic uveal melanoma (Alliance A091201). Clin. Cancer Res..

[19] Carvajal R.D., Piperno-Neumann S., Kapiteijn E., Chapman P.B., Frank S., Joshua A.M., Piulats J.M., Wolter P., Cocquyt V., Chmielowski B. (2018). Selumetinib in Combination With Dacarbazine in Patients With Metastatic Uveal Melanoma: A Phase III, Multicenter, Randomized Trial (SUMIT). J. Clin. Oncol..

[20] Patrone S., Maric I., Rutigliani M., Lanza F., Puntoni M., Banelli B., Rancati S., Angelini G., Amaro A., Ligorio P. (2018). Prognostic value of chromosomal imbalances, gene mutations, and BAP1 expression in uveal melanoma. Genes Chromosomes Cancer.

[21] Yavuzyigitoglu S., Koopmans A.E., Verdijk R.M., Vaarwater J., Eussen B., van Bodegom A., Paridaens D., Kilic E., de Klein A., Rotterdam Ocular Melanoma Study G. (2016). Uveal Melanomas with SF3B1 Mutations: A Distinct Subclass Associated with Late-Onset Metastases. Ophthalmology.

[22] Staby K.M., Gravdal K., Mork S.J., Heegaard S., Vintermyr O.K., Krohn J. (2018). Prognostic impact of chromosomal aberrations and GNAQ, GNA11 and BAP1 mutations in uveal melanoma. Acta Ophthalmol..

[23] Vavvas D., Kim I., Lane A.M., Chaglassian A., Mukai S., Gragoudas E. (2010). Posterior uveal melanoma in young patients treated with proton beam therapy. Retina.

[24] Petrovic A., Bergin C., Schalenbourg A., Goitein G., Zografos L. (2014). Proton therapy for uveal melanoma in 43 juvenile patients: Long-term results. Ophthalmology.

[25] Fry M.V., Augsburger J.J., Correa Z.M. (2019). Clinical Features, Metastasis, and Survival in Patients Younger Than 21 Years With Posterior Uveal Melanoma. JAMA Ophthalmol..

[26] Shields C.L., Kaliki S., Arepalli S., Atalay H.T., Manjandavida F.P., Pieretti G., Shields J.A. (2013). Uveal melanoma in children and teenagers. Saudi J. Ophthalmol..

[27] Al-Jamal R.T., Cassoux N., Desjardins L., Damato B., Konstantinidis L., Coupland S.E., Heimann H., Petrovic A., Zografos L., Schalenbourg A. (2016). The Pediatric Choroidal and Ciliary Body Melanoma Study: A Survey by the European Ophthalmic Oncology Group. Ophthalmology.

[28] Al-Jamal R.T., Kivela T. (2014). Uveal melanoma among Finnish children and young adults. J. AAPOS.

[29] Barr C.C., McLean I.W., Zimmerman L.E. (1981). Uveal melanoma in children and adolescents. Arch. Ophthalmol..

[30] Kaliki S., Shields C.L., Mashayekhi A., Ganesh A., Furuta M., Shields J.A. (2013). Influence of age on prognosis of young patients with uveal melanoma: A matched retrospective cohort study. Eur. J. Ophthalmol..

[31] Dalvin L.A., Salomao D.R., Patel S.V. (2018). Population-based incidence of conjunctival tumours in Olmsted County, Minnesota. Br. J. Ophthalmol..

[32] Larsen A.C. (2016). Conjunctival malignant melanoma in Denmark: Epidemiology, treatment and prognosis with special emphasis on tumorigenesis and genetic profile. Acta Ophthalmol..

[33] Shields C.L., Alset A.E., Boal N.S., Casey M.G., Knapp A.N., Sugarman J.A., Schoen M.A., Gordon P.S., Douglass A.M., Sioufi K. (2017). Conjunctival Tumors in 5002 Cases. Comparative Analysis of Benign Versus Malignant Counterparts. The 2016 James D. Allen Lecture. Am. J. Ophthalmol..

[34] Larsen A.C., Dahl C., Dahmcke C.M., Lade-Keller J., Siersma V.D., Toft P.B., Coupland S.E., Prause J.U., Guldberg P., Heegaard S. (2016). BRAF mutations in conjunctival melanoma: Investigation of incidence, clinicopathological features, prognosis and paired premalignant lesions. Acta Ophthalmol..

[35] Scholz S.L., Cosgarea I., Susskind D., Murali R., Moller I., Reis H., Leonardelli S., Schilling B., Schimming T., Hadaschik E. (2018). NF1 mutations in conjunctival melanoma. Br. J. Cancer.

[36] Balzer B.W.R., Cherepanoff S., Joshua A.M., Giblin M., Conway R.M., Anazodo A.C. (2019). Conjunctival Melanoma in Childhood and Adolescence: A Systematic Review. Ocul. Oncol. Pathol..

[37] Esmaeli B., Rubin M.L., Xu S., Goepfert R.P., Curry J.L., Prieto V.G., Ning J., Tetzlaff M.T. (2019). Greater Tumor Thickness, Ulceration, and Positive Sentinel Lymph Node Are Associated With Worse Prognosis in Patients With Conjunctival Melanoma: Implications for Future AJCC Classifications. Am. J. Surg. Pathol..

[38] Brouwer N.J., Marinkovic M., van Duinen S.G., Bleeker J.C., Jager M.J., Luyten G.P.M. (2018). Treatment of conjunctival melanoma in a Dutch referral centre. Br. J. Ophthalmol..

[39] Kenawy N., Kalirai H., Sacco J.J., Lake S.L., Heegaard S., Larsen A.C., Finger P.T., Milman T., Chin K., Mosci C. (2019). Conjunctival melanoma copy number alterations and correlation with mutation status, tumor features, and clinical outcome. Pigment. Cell Melanoma Res..

[40] Finger P.T., Pavlick A.C. (2019). Checkpoint inhibition immunotherapy for advanced local and systemic conjunctival melanoma: A clinical case series. J. Immunother. Cancer.

[41] Sagiv O., Thakar S.D., Kandl T.J., Ford J., Sniegowski M.C., Hwu W.J., Esmaeli B. (2018). Immunotherapy With Programmed Cell Death 1 Inhibitors for 5 Patients With Conjunctival Melanoma. JAMA Ophthalmol..

[42] Prescher G., Bornfeld N., Hirche H., Horsthemke B., Jockel K.H., Becher R. (1996). Prognostic implications of monosomy 3 in uveal melanoma. Lancet.

[43] Scholz S.L., Moller I., Reis H., Susskind D., van de Nes J.A.P., Leonardelli S., Schilling B., Livingstone E., Schimming T., Paschen A. (2017). Frequent GNAQ, GNA11, and EIF1AX Mutations in Iris Melanoma. Invest. Ophthalmol. Vis. Sci..

[44] Van Poppelen N.M., Vaarwater J., Mudhar H.S., Sisley K., Rennie I.G., Rundle P., Brands T., van den Bosch Q.C.C., Mensink H.W., de Klein A. (2018). Genetic Background of Iris Melanomas and Iris Melanocytic Tumors of Uncertain Malignant Potential. Ophthalmology.

[45] Koopmans A.E., Vaarwater J., Paridaens D., Naus N.C., Kilic E., de Klein A. (2013). Rotterdam Ocular Melanoma Study, g. Patient survival in uveal melanoma is not affected by oncogenic mutations in GNAQ and GNA11. Br. J. Cancer.

[46] Ewens K.G., Kanetsky P.A., Richards-Yutz J., Purrazzella J., Shields C.L., Ganguly T., Ganguly A. (2014). Chromosome 3 status combined with BAP1 and EIF1AX mutation profiles are associated with metastasis in uveal melanoma. Invest. Ophthalmol. Vis. Sci..

[47] Van de Nes J.A., Nelles J., Kreis S., Metz C.H., Hager T., Lohmann D.R., Zeschnigk M. (2016). Comparing the Prognostic Value of BAP1 Mutation Pattern, Chromosome 3 Status, and BAP1 Immunohistochemistry in Uveal Melanoma. Am. J. Surg. Pathol..

[48] Van den Bosch T., van Beek J.G., Vaarwater J., Verdijk R.M., Naus N.C., Paridaens D., de Klein A., Kilic E. (2012). Higher percentage of FISH-determined monosomy 3 and 8q amplification in uveal melanoma cells relate to poor patient prognosis. Invest. Ophthalmol. Vis. Sci..

[49] Hinnebusch A.G. (2014). The scanning mechanism of eukaryotic translation initiation. Annu. Rev. Biochem..

[50] Hinnebusch A.G. (2017). Structural insights into the mechanism of scanning and start codon recognition in eukaryotic translation initiation. Trends Biochem. Sci..

[51] Agrawal N., Akbani R., Aksoy B.A., Ally A., Arachchi H., Asa S.L., Auman J.T., Balasundaram M., Balu S., Baylin S.B. (2014). Integrated genomic characterization of papillary thyroid carcinoma. Cell.

[52] Chaudhuri J., Si K., Maitra U. (1997). Function of eukaryotic translation initiation factor 1A (eIF1A)(formerly called eIF-4C) in initiation of protein synthesis. J. Biol. Chem..

[53] Martin M., Maßhöfer L., Temming P., Rahmann S., Metz C., Bornfeld N., van de Nes J., Klein-Hitpass L., Hinnebusch A.G., Horsthemke B. (2013). Exome sequencing identifies recurrent somatic mutations in EIF1AX and SF3B1 in uveal melanoma with disomy 3. Nat. Genet..

[54] Robertson A.G., Shih J., Yau C., Gibb E.A., Oba J., Mungall K.L., Hess J.M., Uzunangelov V., Walter V., Danilova L. (2017). Integrative analysis identifies four molecular and clinical subsets in uveal melanoma. Cancer Cell.

[55] Lee S., Liu B., Lee S., Huang S.-X., Shen B., Qian S.-B. (2012). Global mapping of translation initiation sites in mammalian cells at single-nucleotide resolution. Proc. Natl. Acad. Sci. USA.

[56] Violanti S.S., Bononi I., Gallenga C.E., Martini F., Tognon M., Perri P. (2019). New insights into molecular oncogenesis and therapy of uveal melanoma. Cancers.

[57] Seiler M., Peng S., Agrawal A.A., Palacino J., Teng T., Zhu P., Smith P.G., Caesar-Johnson S.J., Demchok J.A., Felau I. (2018). Somatic mutational landscape of splicing factor genes and their functional consequences across 33 cancer types. Cell Rep..

[58] Cretu C., Schmitzová J., Ponce-Salvatierra A., Dybkov O., De Laurentiis E.I., Sharma K., Will C.L., Urlaub H., Lührmann R., Pena V. (2016). Molecular architecture of SF3b and structural consequences of its cancer-related mutations. Mol. Cell.

[59] Gozani O., Potashkin J., Reed R. (1998). A potential role for U2AF-SAP 155 interactions in recruiting U2 snRNP to the branch site. Mol. Cell. Biol..

[60] Will C.L., Urlaub H., Achsel T., Gentzel M., Wilm M., Lührmann R. (2002). Characterization of novel SF3b and 17S U2 snRNP proteins, including a human Prp5p homologue and an SF3b DEAD-box protein. EMBO J..

[61] Darman R.B., Seiler M., Agrawal A.A., Lim K.H., Peng S., Aird D., Bailey S.L., Bhavsar E.B., Chan B., Colla S. (2015). Cancer-associated SF3B1 hotspot mutations induce cryptic 3′ splice site selection through use of a different branch point. Cell Rep..

[62] Alsafadi S., Houy A., Battistella A., Popova T., Wassef M., Henry E., Tirode F., Constantinou A., Piperno-Neumann S., Roman-Roman S. (2016). Cancer-associated SF3B1 mutations affect alternative splicing by promoting alternative branchpoint usage. Nat. Commun..

[63] Furney S.J., Pedersen M., Gentien D., Dumont A.G., Rapinat A., Desjardins L., Turajlic S., Piperno-Neumann S., de la Grange P., Roman-Roman S. (2013). SF3B1 mutations are associated with alternative splicing in uveal melanoma. Cancer Discov..

[64] Harbour J.W., Roberson E.D., Anbunathan H., Onken M.D., Worley L.A., Bowcock A.M. (2013). Recurrent mutations at codon 625 of the splicing factor SF3B1 in uveal melanoma. Nat. Genet..

[65] Mensink H.W., Vaarwater J., de Keizer R.J., de Wolff-Rouendaal D., Mooy C.M., de Klein A., Paridaens D. (2011). Chromosomal aberrations in iris melanomas. Br. J. Ophthalmol..

[66] Fabian I.D., Thaung C., AlHarby L., Sisley K., Mudhar H.S., Doherty R.E., Stacey A.W., Arora A.K., Cohen V.M.L., Sagoo M.S. (2017). Late Solitary Extraocular Recurrence From Previously Resected Iris Melanoma. Am. J. Ophthalmol..

[67] Yavuzyigitoglu S., Drabarek W., Smit K.N., van Poppelen N., Koopmans A.E., Vaarwater J., Brands T., Eussen B., Dubbink H.J., van Riet J. (2017). Correlation of Gene Mutation Status with Copy Number Profile in Uveal Melanoma. Ophthalmology.

[68] Johansson P., Aoude L.G., Wadt K., Glasson W.J., Warrier S.K., Hewitt A.W., Kiilgaard J.F., Heegaard S., Isaacs T., Franchina M. (2016). Deep sequencing of uveal melanoma identifies a recurrent mutation in PLCB4. Oncotarget.

[69] Field M.G., Durante M.A., Anbunathan H., Cai L.Z., Decatur C.L., Bowcock A.M., Kurtenbach S., Harbour J.W. (2018). Punctuated evolution of canonical genomic aberrations in uveal melanoma. Nat. Commun..

[70] Van Poppelen N.M., Drabarek W., Smit K.N., Vaarwater J., Brands T., Paridaens D., Kilic E., de Klein A. (2019). SRSF2 Mutations in Uveal Melanoma: A Preference for In-Frame Deletions?. Cancers.

[71] Davies H., Bignell G.R., Cox C., Stephens P., Edkins S., Clegg S., Teague J., Woffendin H., Garnett M.J., Bottomley W. (2002). Mutations of the BRAF gene in human cancer. Nature.

[72] Swaminathan S.S., Field M.G., Sant D., Wang G., Galor A., Dubovy S.R., Harbour J.W., Karp C.L. (2017). Molecular Characteristics of Conjunctival Melanoma Using Whole-Exome Sequencing. JAMA Ophthalmol..

[73] Koopmans A.E., Ober K., Dubbink H.J., Paridaens D., Naus N.C., Belunek S., Krist B., Post E., Zwarthoff E.C., de Klein A. (2014). Prevalence and implications of TERT promoter mutation in uveal and conjunctival melanoma and in benign and premalignant conjunctival melanocytic lesions. Invest. Ophthalmol. Vis. Sci..

[74] Van Ipenburg J.N.N., Dubbink H.J., van Ginderdeuren R., Missotten G.S., Paridaens D., Verdijk R.M. (2020). Prognostic value of TERT promoter mutations in conjunctival melanomas in addition to clinicopathological features. Br. J. Ophthalmol..

[75] Gupta M.P., Lane A.M., DeAngelis M.M., Mayne K., Crabtree M., Gragoudas E.S., Kim I.K. (2015). Clinical Characteristics of Uveal Melanoma in Patients With Germline BAP1 Mutations. JAMA Ophthalmol..

[76] Aoude L.G., Vajdic C.M., Kricker A., Armstrong B., Hayward N.K. (2013). Prevalence of germline BAP1 mutation in a population-based sample of uveal melanoma cases. Pigment. Cell Melanoma Res..

[77] Turunen J.A., Markkinen S., Wilska R., Saarinen S., Raivio V., Tall M., Lehesjoki A.E., Kivela T.T. (2016). BAP1 Germline Mutations in Finnish Patients with Uveal Melanoma. Ophthalmology.

[78] Popova T., Hebert L., Jacquemin V., Gad S., Caux-Moncoutier V., Dubois-d’Enghien C., Richaudeau B., Renaudin X., Sellers J., Nicolas A. (2013). Germline BAP1 mutations predispose to renal cell carcinomas. Am. J. Hum. Genet..

[79] Rai K., Pilarski R., Boru G., Rehman M., Saqr A.H., Massengill J.B., Singh A., Marino M.J., Davidorf F.H., Cebulla C.M. (2017). Germline BAP1 alterations in familial uveal melanoma. Genes Chromosomes Cancer.

[80] Chau C., van Doorn R., van Poppelen N.M., van der Stoep N., Mensenkamp A.R., Sijmons R.H., van Paassen B.W., van den Ouweland A.M.W., Naus N.C., van der Hout A.H. (2019). Families with BAP1-Tumor Predisposition Syndrome in The Netherlands: Path to Identification and a Proposal for Genetic Screening Guidelines. Cancers.

[81] Ewens K.G., Lalonde E., Richards-Yutz J., Shields C.L., Ganguly A. (2018). Comparison of Germline versus Somatic BAP1 Mutations for Risk of Metastasis in Uveal Melanoma. BMC Cancer.

[82] Njauw C.N., Kim I., Piris A., Gabree M., Taylor M., Lane A.M., DeAngelis M.M., Gragoudas E., Duncan L.M., Tsao H. (2012). Germline BAP1 inactivation is preferentially associated with metastatic ocular melanoma and cutaneous-ocular melanoma families. PLoS ONE.

[83] Walpole S., Pritchard A.L., Cebulla C.M., Pilarski R., Stautberg M., Davidorf F.H., de la Fouchardiere A., Cabaret O., Golmard L., Stoppa-Lyonnet D. (2018). Comprehensive Study of the Clinical Phenotype of Germline BAP1 Variant-Carrying Families Worldwide. J. Natl. Cancer Inst..

[84] Massengill J.B., Sample K.M., Pilarski R., McElroy J., Davidorf F.H., Cebulla C.M., Abdel-Rahman M.H. (2018). Analysis of the exome aggregation consortium (ExAC) database suggests that the BAP1-tumor predisposition syndrome is underreported in cancer patients. Genes Chromosomes Cancer.

[85] Hajkova N., Hojny J., Nemejcova K., Dundr P., Ulrych J., Jirsova K., Glezgova J., Ticha I. (2018). Germline mutation in the TP53 gene in uveal melanoma. Sci Rep..

[86] Jay M., McCartney A.C. (1993). Familial malignant melanoma of the uvea and p53: A Victorian detective story. Surv. Ophthalmol..

[87] Seddon J.M., Albert D.M., Lavin P.T., Robinson N. (1983). A prognostic factor study of disease-free interval and survival following enucleation for uveal melanoma. Arch. Ophthalmol..

[88] Folberg R., Rummelt V., Parys-Van Ginderdeuren R., Hwang T., Woolson R.F., Pe’er J., Gruman L.M. (1993). The prognostic value of tumor blood vessel morphology in primary uveal melanoma. Ophthalmology.

[89] Seregard S., Kock E. (1995). Prognostic indicators following enucleation for posterior uveal melanoma. A multivariate analysis of long-term survival with minimized loss to follow-up. Acta Ophthalmol. Scand..

[90] Zloto O., Pe’er J., Frenkel S. (2013). Gender differences in clinical presentation and prognosis of uveal melanoma. Invest. Ophthalmol. Vis. Sci..

[91] Ewens K.G., Kanetsky P.A., Richards-Yutz J., Al-Dahmash S., De Luca M.C., Bianciotto C.G., Shields C.L., Ganguly A. (2013). Genomic profile of 320 uveal melanoma cases: Chromosome 8p-loss and metastatic outcome. Investig. Ophthalmol. Vis. Sci..

[92] Shields C.L., Shields J.A., Eagle R.C., De Potter P., Menduke H. (1991). Uveal melanoma and pregnancy. A report of 16 cases. Ophthalmology.

[93] Kilic E., Naus N.C., van Gils W., Klaver C.C., van Til M.E., Verbiest M.M., Stijnen T., Mooy C.M., Paridaens D., Beverloo H.B. (2005). Concurrent loss of chromosome arm 1p and chromosome 3 predicts a decreased disease-free survival in uveal melanoma patients. Investig. Ophthalmol. Vis. Sci..

[94] Prescher G., Bornfeld N., Horsthemke B., Becher R. (1992). Chromosomal aberrations defining uveal melanoma of poor prognosis. Lancet.

[95] Sisley K., Rennie I.G., Parsons M.A., Jacques R., Hammond D.W., Bell S.M., Potter A.M., Rees R.C. (1997). Abnormalities of chromosomes 3 and 8 in posterior uveal melanoma correlate with prognosis. Genes Chromosomes Cancer.

[96] Patel K.A., Edmondson N.D., Talbot F., Parsons M.A., Rennie I.G., Sisley K. (2001). Prediction of prognosis in patients with uveal melanoma using fluorescence in situ hybridisation. Br. J. Ophthalmol..

[97] Shields C.L., Ganguly A., Bianciotto C.G., Turaka K., Tavallali A., Shields J.A. (2011). Prognosis of uveal melanoma in 500 cases using genetic testing of fine-needle aspiration biopsy specimens. Ophthalmology.

[98] Onken M.D., Worley L.A., Person E., Char D.H., Bowcock A.M., Harbour J.W. (2007). Loss of heterozygosity of chromosome 3 detected with single nucleotide polymorphisms is superior to monosomy 3 for predicting metastasis in uveal melanoma. Clin. Cancer Res..

[99] Harbour J.W., Onken M.D., Roberson E.D., Duan S., Cao L., Worley L.A., Council M.L., Matatall K.A., Helms C., Bowcock A.M. (2010). Frequent mutation of BAP1 in metastasizing uveal melanomas. Science.

[100] Shah A.A., Bourne T.D., Murali R. (2013). BAP1 protein loss by immunohistochemistry: A potentially useful tool for prognostic prediction in patients with uveal melanoma. Pathology.

[101] Kalirai H., Dodson A., Faqir S., Damato B.E., Coupland S.E. (2014). Lack of BAP1 protein expression in uveal melanoma is associated with increased metastatic risk and has utility in routine prognostic testing. Br. J. Cancer.

[102] Koopmans A.E., Verdijk R.M., Brouwer R.W., van den Bosch T.P., van den Berg M.M., Vaarwater J., Kockx C.E., Paridaens D., Naus N.C., Nellist M. (2014). Clinical significance of immunohistochemistry for detection of BAP1 mutations in uveal melanoma. Mod. Pathol..

[103] Worley L.A., Onken M.D., Person E., Robirds D., Branson J., Char D.H., Perry A., Harbour J.W. (2007). Transcriptomic versus chromosomal prognostic markers and clinical outcome in uveal melanoma. Clin. Cancer Res..

[104] Petrausch U., Martus P., Tonnies H., Bechrakis N.E., Lenze D., Wansel S., Hummel M., Bornfeld N., Thiel E., Foerster M.H. (2008). Significance of gene expression analysis in uveal melanoma in comparison to standard risk factors for risk assessment of subsequent metastases. Eye (London).

[105] Tschentscher F., Husing J., Holter T., Kruse E., Dresen I.G., Jockel K.H., Anastassiou G., Schilling H., Bornfeld N., Horsthemke B. (2003). Tumor classification based on gene expression profiling shows that uveal melanomas with and without monosomy 3 represent two distinct entities. Cancer Res..

[106] Worley L.A., Long M.D., Onken M.D., Harbour J.W. (2008). Micro-RNAs associated with metastasis in uveal melanoma identified by multiplexed microarray profiling. Melanoma Res..

[107] Falzone L., Romano G.L., Salemi R., Bucolo C., Tomasello B., Lupo G., Anfuso C.D., Spandidos D.A., Libra M., Candido S. (2019). Prognostic significance of deregulated microRNAs in uveal melanomas. Mol. Med. Rep..

[108] Geisse L.J., Robertson D.M. (1985). Iris melanomas. Am. J. Ophthalmol..

[109] Paridaens A.D., Minassian D.C., McCartney A.C., Hungerford J.L. (1994). Prognostic factors in primary malignant melanoma of the conjunctiva: A clinicopathological study of 256 cases. Br. J. Ophthalmol..

[110] Shields C.L., Kaliki S., Shah S.U., Luo W., Furuta M., Shields J.A. (2012). Iris melanoma: Features and prognosis in 317 children and adults. J. AAPOS.

[111] Khan S., Finger P.T., Yu G.P., Razzaq L., Jager M.J., de Keizer R.J., Sandkull P., Seregard S., Gologorsky D., Schefler A.C. (2012). Clinical and pathologic characteristics of biopsy-proven iris melanoma: A multicenter international study. Arch. Ophthalmol..

[112] Shields C.L., Shah S.U., Bianciotto C.G., Emrich J., Komarnicky L., Shields J.A. (2013). Iris melanoma management with iodine-125 plaque radiotherapy in 144 patients: Impact of melanoma-related glaucoma on outcomes. Ophthalmology.

[113] Oxenreiter M.M., Lane A.M., Jain P., Kim I.K., Gragoudas E.S. (2019). Conservative management of suspicious melanocytic lesions of the iris. Graefes Arch. Clin. Exp. Ophthalmol..

[114] Cheng J., Holland-Letz T., Wallwiener M., Surowy H., Cuk K., Schott S., Trumpp A., Pantel K., Sohn C., Schneeweiss A. (2018). Circulating free DNA integrity and concentration as independent prognostic markers in metastatic breast cancer. Breast. Cancer Res. Treat..

[115] Xiang Z., Wan R., Zou B., Qi X., Huang Q., Kumar S., Pitman J.L., Zhou G., Song Q. (2018). Highly sensitive and specific real-time PCR by employing serial invasive reaction as a sequence identifier for quantifying EGFR mutation abundance in cfDNA. Anal. Bioanal. Chem..

[116] Missotten G.S., Keijser S., De Keizer R.J., De Wolff-Rouendaal D. (2005). Conjunctival melanoma in the Netherlands: A nationwide study. Invest. Ophthalmol. Vis. Sci..

[117] Esmaeli B., Roberts D., Ross M., Fellman M., Cruz H., Kim S.K., Prieto V.G. (2012). Histologic features of conjunctival melanoma predictive of metastasis and death (an American Ophthalmological thesis). Trans. Am. Ophthalmol. Soc..

[118] Heindl L.M., Hofmann-Rummelt C., Adler W., Bosch J.J., Holbach L.M., Naumann G.O., Kruse F.E., Cursiefen C. (2011). Prognostic significance of tumor-associated lymphangiogenesis in malignant melanomas of the conjunctiva. Ophthalmology.

[119] Sheng X., Li S., Chi Z., Si L., Cui C., Mao L., Lian B., Tang B., Wang X., Yan X. (2015). Prognostic factors for conjunctival melanoma: A study in ethnic Chinese patients. Br. J. Ophthalmol..

[120] Mor J.M., Heindl L.M. (2017). Systemic BRAF/MEK Inhibitors as a Potential Treatment Option in Metastatic Conjunctival Melanoma. Ocul. Oncol. Pathol..

[121] Wei L., Wu W., Han L., Yu W., Du Y. (2018). A quantitative analysis of the potential biomarkers of non-small cell lung cancer by circulating cell-free DNA. Oncol. Lett..

[122] Takeshita T., Yamamoto Y., Yamamoto-Ibusuki M., Tomiguchi M., Sueta A., Murakami K., Iwase H. (2018). Clinical significance of plasma cell-free DNA mutations in PIK3CA, AKT1, and ESR1 gene according to treatment lines in ER-positive breast cancer. Mol. Cancer.

[123] Allard W.J., Matera J., Miller M.C., Repollet M., Connelly M.C., Rao C., Tibbe A.G., Uhr J.W., Terstappen L.W. (2004). Tumor cells circulate in the peripheral blood of all major carcinomas but not in healthy subjects or patients with nonmalignant diseases. Clin. Cancer Res..

[124] Anand K., Roszik J., Gombos D., Upshaw J., Sarli V., Meas S., Lucci A., Hall C., Patel S. (2019). Pilot Study of Circulating Tumor Cells in Early-Stage and Metastatic Uveal Melanoma. Cancers.

[125] Bidard F.C., Madic J., Mariani P., Piperno-Neumann S., Rampanou A., Servois V., Cassoux N., Desjardins L., Milder M., Vaucher I. (2014). Detection rate and prognostic value of circulating tumor cells and circulating tumor DNA in metastatic uveal melanoma. Int. J. Cancer.

[126] Aaron B., Timothy I., Muhammad A.K., James B.F., Richard A., Fred K.C., Michelle R.P., Kyle Y., Jaqueline B., Tersia V. (2018). Clinical Application of Circulating Tumor Cells and Circulating Tumor DNA in Uveal Melanoma. JCO Precis. Oncol..

[127] Schuster R., Bechrakis N.E., Stroux A., Busse A., Schmittel A., Scheibenbogen C., Thiel E., Foerster M.H., Keilholz U. (2007). Circulating tumor cells as prognostic factor for distant metastases and survival in patients with primary uveal melanoma. Clin. Cancer Res..

[128] Pinzani P., Mazzini C., Salvianti F., Massi D., Grifoni R., Paoletti C., Ucci F., Molinara E., Orlando C., Pazzagli M. (2010). Tyrosinase mRNA levels in the blood of uveal melanoma patients: Correlation with the number of circulating tumor cells and tumor progression. Melanoma Res..

[129] Bebelman M.P., Smit M.J., Pegtel D.M., Baglio S.R. (2018). Biogenesis and function of extracellular vesicles in cancer. Pharmacol. Ther..

[130] Eldh M., Olofsson Bagge R., Lasser C., Svanvik J., Sjostrand M., Mattsson J., Lindner P., Choi D.S., Gho Y.S., Lotvall J. (2014). MicroRNA in exosomes isolated directly from the liver circulation in patients with metastatic uveal melanoma. BMC Cancer.

[131] Kivela T., Eskelin S., Makitie T., Summanen P. (2001). Exudative retinal detachment from malignant uveal melanoma: Predictors and prognostic significance. Investig. Ophthalmol. Vis. Sci..

